# The Physical Activity Environment, Nature-Relatedness and Wellbeing

**DOI:** 10.3390/ijerph22020299

**Published:** 2025-02-17

**Authors:** Josh Furjes-Crawshaw, Ihirangi Heke, Tim Jowett, Nancy J. Rehrer

**Affiliations:** 1 School of Physical Education Sport & Exercise Sciences, University of Otago, Dunedin 9054, New Zealand; joshfurjes@gmail.com; 2 Waikato Tainui, 279 School Rd. Te Arai, Auckland 0974, New Zealand; ihi.heke@xtra.co.nz; 3 Department of Mathematics and Statistics, University of Otago, P.O. Box 56, Dunedin 9054, New Zealand; timothy.jowett@otago.ac.nz

**Keywords:** environment, exercise, green space, health, nature-relatedness

## Abstract

This study explored the relationship between the physical activity (PA) environment, nature affinity and wellbeing. An online survey was used incorporating the Nature-Relatedness Scale (NR-6), EQ-5D health questionnaire, WHO-5 wellbeing questionnaire and International Physical Activity Questionnaire—Short Form (IPAQ-SF), with additional questions on PA environment and connection to place (*n* = 179). Statistical analyses were conducted using generalised linear mixed effects and quantile regression. PA in nature was correlated with wellbeing, with each additional bout of PA in nature associated with an increase in EQ-5D score of 3.13 and an increase in WHO-5 score of 5.62, (*p* = 0.0058, η_p_^2^ = 0.074; *p* < 0.0001, η_p_^2^ = 0.089, resp. (medium effect sizes)). PA indoors was also positively associated with physical and psychological wellbeing (*p* = 0.0192, η_p_^2^ ₌ 0.018; *p* = 0.0009, η_p_^2^ = 0.03, resp. (small effect sizes)), but PA in nature had a greater effect than PA indoors on both physical (*p* = 0.008) and psychological wellbeing (*p* = 0.001). There was a significant interaction between nature-relatedness and PA in nature on wellbeing (*p* = 0.0002), indicating a difference in the association between nature-relatedness and both physical and psychological wellbeing, i.e., there was a greater effect of PA in nature on wellbeing in those with greater nature-relatedness. Nature-relatedness was also associated with physical activity in nature (*p* ≤ 0.0001).

## 1. Introduction

### 1.1. Background

Regular physical activity (PA) has been well documented to provide a range of physiological and psychological benefits that reduce the risk of a number of non-communicable diseases, morbidity and mortality [1,2,3] and improve mental health [4,5] as well as academic performance and cognition [6]. The World Health Organisation recommends 150–300 min of moderate intensity PA, or 75–150 min of vigorous PA, per week to reduce the ill effects of sedentary behaviour on health [7]. The physical environment can influence PA. The quality of outdoor (built) environments acts to impose barriers, e.g., low-quality environments can discourage [8], or encourage PA. Environmental factors can positively influence physical activity, in particular, natural, green spaces [9,10]. Living in close proximity to green spaces can increase the likelihood of meeting PA recommendations [11,12]. Exposure to natural environments has also been associated with positive mental health outcomes [13,14,15]. Living close to green space is correlated with not only greater psychological wellbeing but also a reduction in a number of chronic disease states [12,16] and all-cause mortality [17,18].

E.O. Wilson posed that humans’ affinity for the natural world is innate, humans are deeply interconnected with all other living things, and contact with nature is a basic human need required for humans to thrive [19,20]. One method designed to quantify one’s innate connection to nature is to assess nature-relatedness [21]. Those who express greater nature-relatedness demonstrate greater eudaimonic wellbeing than those without [22]. Furthermore, those who demonstrate nature-relatedness are likely to feel happier and connected with the environment and experience reductions in anxiety and more satisfaction in life [23,24]. It follows that conducting PA in natural environments may bring more benefits than when PA is conducted elsewhere. There is some evidence of this synergistic effect. Mitchell [25] observed a reduced risk of poor mental health when PA was conducted in natural environments (open space/park, woodland/forest) in Scotland. Pasanen et al. [26] similarly found, in Finland, better emotional wellbeing when PA was conducted in nature.

It is unknown, in the New Zealand context, if PA in nature is associated with physical and/or psychological wellbeing similar to or different from that in other environments. It is also unknown if nature-relatedness is positively associated with increased PA in nature and enhanced physical and/or psychological wellbeing.

Intertwined with nature-relatedness is one’s sense of connection to place, which can be defined as the bonding that occurs between individuals and their meaningful environments [27]. Connection to place has been considered by some to be emotional and fulfils a fundamental human need [28,29]. This has relevance for the indigenous inhabitants of New Zealand (Māori). In the Māori traditional worldview, the interconnectedness between all living things is acknowledged [30]. The traditional Māori concept of tūrangawaewae or “place to stand” gives importance to the power of connectedness to a place and aligns with these more recent understandings [31]. Connection to place can also be enriched by knowing the stories from the past that increase a sense of belonging to a larger family or genealogy that includes the environment [32]. Whether having this type of connection to place enhances wellbeing, and being physically active in nature, is unknown.

### 1.2. Research Objectives

The present study was designed to explore the relationship between (1) PA environment (nature, built outdoors, indoors) and perceived wellbeing and (2) nature-relatedness or connection to place and PA in nature and perceived wellbeing. It is hypothesised that PA in nature is correlated with both physical and psychological wellbeing to a greater extent than in other environments and that nature-relatedness and connection to place are both correlated with PA in nature and enhanced psychological and physical wellbeing.

## 2. Materials and Methods

### 2.1. Study Design

This study was observational. An online, self-report survey combining previously published questionnaires and self-designed questions was utilised. Recruitment was conducted by university websites and emails, social media, posters and word of mouth. The inclusion criteria included adults (18+ years old) living in New Zealand with access to the internet. Data were collected between 1 August 2021 and 31 October 2021. This study was approved by the University of Otago Ethical Committee (reference number: D21/207; 9 July 2021), and online informed consent was required prior to completing the survey.

### 2.2. Respondents and Sociodemographic Characteristics

Two hundred and twenty-one individuals accessed the survey, with 19% (*n* = 42) excluded from analyses as they failed to meet the inclusion criteria. Exclusion from the final analyses was primarily due to incomplete (<80%) surveys, 14% (*n* = 31). Additional reasons for exclusion included missing necessary content (e.g., missing one or more measures of wellbeing), 3.2% (*n* = 7); not currently living in New Zealand, 1.4% (*n* = 3); or not being at least 18 years of age, 0.5% (*n* = 1). Thus, 179 individual surveys were included in the analyses. Not all respondents answered all questions; thus, the results presented are from the respondents that answered each specific question.

The sociodemographic characteristics of those whose data were included in the analysis are presented in Table 1. Just over half of the respondents were female, and the majority were of New Zealand European ethnicity, with the next most common ethnicities being Māori, followed by Pasifika. Just under two-thirds of the respondents were single/living alone; the rest were either married or living with a partner. Around three-quarters of the respondents had gained at least an undergraduate qualification, with over half of the respondents being employed and around one-third currently enrolled in study. A small but not insignificant number of respondents indicated that they had a disability that impaired physical activity.

### 2.3. Data Collection

The survey, implemented via Qualtrics^®^, was able to be accessed either via a web link or a QR code. There was no limitation as to the number of individuals able to complete the survey; sample size was only limited by self-selection over the given timeframe. The “prevent multiple submissions” option was used in which duplicate submissions are recognised. No duplicates were found. As this research was exploratory in nature, combining several questionnaires and additional novel questions, no a priori power analysis was conducted.

#### Survey Instrument

The outcome measures that were used for this research came from the following sections of the survey in the following order: sociodemographics, nature-relatedness, perceived general wellbeing, perceived psychological wellbeing, type of environment physically active in and connection to place. Sociodemographics, including gender, ethnicity, age, education level, employment and marital status, used categories identical to the most recent New Zealand Census.

PA status was measured with the International Physical Activity Questionnaire—Short Form (IPAQ-SF) [33] due to its wide usage, excellent test–retest reliability and validity with accelerometer data. Typical weekly PA frequency in different environments was determined as described by Pasanen et al. [26]; briefly, bouts of PA of 20 min or longer were counted, and the percentage of all weekly bouts in each environment category was estimated to calculate PA frequency in each environment. Their categories included Indoors, Outdoors in a Built Setting (e.g., sport specific grounds, city streets/cycle lanes, outdoor swimming pool) and Nature (natural environments, including parks, forests, etc.). In the present study, Nature was broken down into three subcategories: Nature-Constructed (e.g., formal gardens, ski fields, farmers’ fields, exotic pine forests), Nature-Wilderness/Native Bush and Nature-Water (e.g., ocean, lakes, rivers and streams); however, they were also combined as one Nature category for comparative analysis.

Physical wellbeing was assessed via the EQ-5D Health visual analogue scale in which participants indicate how good or bad their health state is at the present moment [34]. The scale ranges from 100, described as ‘best imaginable health state’, to zero, described as ‘worst imaginable health state’, where participants slide the bar to their perceived current health state on the day. The EQ-5D Health Questionnaire was chosen due to its validity, feasibility, redistribution properties and a reduced ceiling in comparison with previous models [35]. Perceived psychological wellbeing was measured by the World Health Organisation (WHO-5) Wellbeing Questionnaire, consisting of five questions concerning one’s psychological wellbeing over the previous two weeks [36]. This questionnaire was chosen due to its high clinimetric validity and has been shown to have good applicability across various fields [37]. The questionnaire contains key items, mood (“I feel cheerful and in good spirits”, “I feel calm and relaxed”), interests (“My daily life is filled with things that interest me”) and energy (“I feel active and vigorous”, “I wake up feeling fresh and rested”), all of which are important features relating to affective states, a core component of mental health and wellbeing [38]. A five-point Likert scale was used to assess each of the five questions, ranging from ‘strongly agree’ (5) to ‘strongly disagree’ (1). A key factor in selecting WHO-5 is that it assesses psychological wellbeing, both positive and negative, not merely an absence of symptoms associated with mental health disorders, i.e., has no ceiling effect.

Nature-relatedness was measured by the NR-6 scale as developed and validated by Nisbet and Zelenski [39]. It is a shortened, six-question version of the 21-question scale, which was developed to assess a person’s interest in, fascination with and desire for nature contact [21]. This measure was chosen for its good internal consistency, its temporal stability and being relatively brief. As in NR-6, the scores for each question ranged from 1 (best) to 5 (worst); for the analyses, these were inverted, such that 5 was best and 1 was worst, and a mean of all six questions was calculated for an individual’s overall score.

Connection to place was assessed by a unique set of questions developed by the authors to evaluate if one had an affinity for a particular place and if constructs of familiarity influenced behaviour. Some questions in the survey were unrelated to the main research questions presented here and are not included (complete survey, including self-design questions, is provided in Supplementary Material).

### 2.4. Data Analysis

Completion of at least 80% of the questions and all measures of wellbeing was required to be included in the data analysis. NR-6 and WHO-5 data were collected on a 5-point Likert scale, where 1 was the best possible and 5 was the worst possible rating; the data were inverted so that 1 was the worst and 5 was the best. The WHO-5 raw scores consisted of adding up the ratings from five questions, thus ranging from 5 to 25, and then multiplying by 4 to obtain a standardised percentage score, where 20 indicated very low psychological wellbeing and 100 represented maximal psychological wellbeing [36]. For descriptive analyses, the WHO-5 and NR-6 data were split into 3-category variables for descriptive analysis [40]. For example, participants’ responses that chose ‘somewhat disagree’ or ‘strongly disagree’ were classified as a ‘disagree’ response. Responses of ‘neither agree nor disagree’ were classified as a ‘neutral’ response. Participants’ response selections of ‘somewhat agree’ or ‘strongly agree’ were classified as an ‘agree’ response.

From the IPAQ-SF, MET- min per week was calculated. Those with PA levels of 3000 MET-min per week or more were categorised as highly active; less than 3000 but more than 600 MET-min per week were categorised as moderately active; and less than 600 MET-min per week were categorised as low active [33]. To assess the respondents’ physical activity in different environments, PA measures were quantified by merging two variables, one estimating weekly frequency of PA bouts and the other recording locations where PA takes place [26]. For example, if a respondent claimed to have exercised four times a week, 50 percent indoors and 50 percent in nature, the new variables PA indoors and PA nature would both equal 2 (4 × 0.5). Data collected for connection to place were either a yes (1) or no (2) for each question, splitting the participants into one of two groups.

The data were analysed with R, version 4.4.1 [41]. The associations between the outcome variables General Wellbeing and Psychological Wellbeing and predictor variables Physical Activity in Nature, Nature-relatedness and Connection to Place were investigated by fitting a multivariate generalised linear mixed effects model (GLMEM) using the glmmTMB package [42], assuming a beta distribution with a logit link function for the response. To control for potential confounding effects, the model included the covariates Gender, Age, MET-min/wk (IPAQ) and Disability as well as their two-way interactions. To enable model stability, MET-min/wk data were centred and scaled (raw values divided by SD). To enable model fitting using the beta distribution, which models data in the range between 0 and 1 (not including 0 and 1), the two wellbeing response variables, which in the original form are on a scale between 0 and 100, were transformed by dividing the original values by 100. This is a linear transformation that has no effect on statistical significance. After the initial transformation, four observations for each response variable were equal to 1, which were then adjusted slightly by subtracting 0.001 to keep the values within the required range of the beta distribution. This adjustment is so small that it has essentially no effect on the results.

Model validation (model fit, dispersion and outliers) was performed using the DHARMa package (version 0.4.7) [43]. All tests showed that the model fitted the data and that the distributional assumptions were valid. The performance package (version 2021) [44] was used to measure the percentage of variance explained (r^2^) by the model and the effects of interest using Ferrari and Cribari-Neto [45], which showed that the r^2^ for the model was 0.60. Trends, *p*-values and effect size were assessed by averaging across covariates and predictor variables. The emmeans (version 1.10.5) [46] and multcomp (version 1.4-28) [47] packages were used to quantify effect size and perform statistical tests for the GLMEM model. Unless specified otherwise, the emmeans trend plots were evaluated by averaging across the levels of other predictor variables not included in the plots. Estimated trends were transformed to the response scale evaluated at the mean value of the variable of interest averaged across other predictors. The confidence intervals for these trends were constructed using the single-fit bootstrap method [48].

A quantile regression model was fitted using the quantreg R package (version 6.00) [49] to investigate the association between predictor variables: Nature-relatedness and Connection to Place with Physical Activity in Nature as the outcome variable. Covariates, including Age, Gender and Disability, were also included in the model to control for potential confounding effects. Quantile regression was used because the skewed nature of the response variable prevented valid inference using conventional parametric modelling.

## 3. Results

### 3.1. Wellbeing

As indicated by the EQ-5D visual analogue scale, which ranged from 0 to 100, worst to best health state, the mean respondents perceived general wellbeing was 76.2 ± 14.9. No one rated their health less than 20, and when partitioning the responses in 10% groupings, the largest group was between 80 and 89, with 55% of the respondents rating their health between 80 and 100.

The WHO-5 measure of psychological wellbeing questions indicated good overall mental health. The raw mean score of the five questions was 18.4 ± 3.6, and the standardised percentage was 73.6 ± 14.4 (complete WHO-5 results per question provided in Supplementary Material Table S1).

### 3.2. Physical Activity Status (IPAQ-SF)

Over 50% of the respondents had a high physical activity level as determined by the IPAQ-SF (Table 2). The mean activity level was 4547 ± 4981 MET-min/week. The vast majority (96%) performed PA with others, and 45% performed their PA with animals, one or more times per week.

### 3.3. Physical Activity Environment

The most frequented environment for PA was Indoors (Table 3). Eighty-three percent of the respondents exercised indoors one or more times per week (>20 min/bout). The second most frequented environment for PA was Nature, with 66% conducting PA in this environment at least once a week. Within this category, Nature-Constructed was the most accessed for PA, with 50% utilising this environment and 50% conducting PA in Nature-Wilderness/Native Bush. Nature-Water was the least frequented environment for PA, with just 25% conducting PA in this environment. Outdoors Built was least frequented, but 72% of the respondents conducted PA in this environment at least once a week.

### 3.4. Physical Activity Environment and Wellbeing

**Figure 1:**
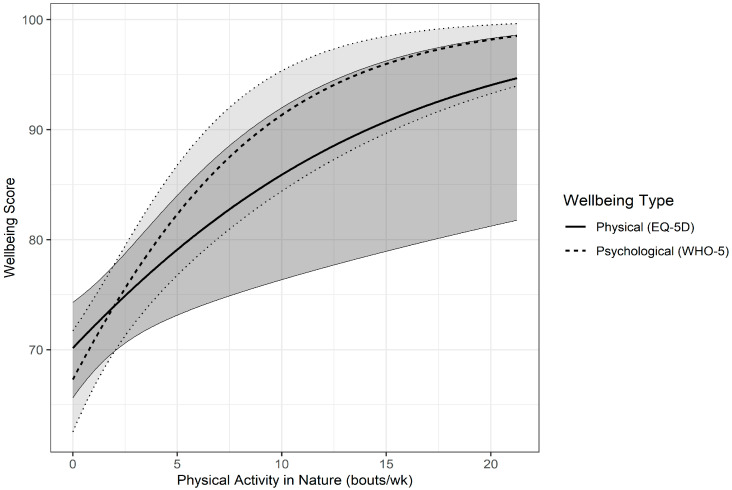
The association between the predictor variable, Physical Activity in Nature, and the outcome variables, mean Physical (EQ-5D) and Psychological (WHO-5) Wellbeing scores, estimated from the GLMEM model. The grey areas designate the 95% confidence bands.

From the fitted GLMEM, a significant interaction of PA in nature and wellbeing was identified (ChiSq(1) = 7.83, *p* = 0.005), which shows the magnitude of the association between physical activity and wellbeing is different for each wellbeing type. There was a positive and statistically significant association between PA in nature and physical wellbeing as determined by the EQ-5D (z = 2.76, *p* = 0.0058) (Figure 1). Every one bout increase in PA in nature was associated with a mean 3.13 unit increase in physical wellbeing score, with a η_p_^2^ of 0.074 (medium effect size).

There was also a positive and statistically significant (z = 4.52, *p* < 0.0001) association between PA in nature and psychological wellbeing as determined by the WHO-5 (Figure 1). Every one bout increase in PA in nature was associated with a mean 5.62 unit increase in psychological wellbeing score, with a η_p_^2^ of 0.089 (medium effect size).

**Figure 2:**
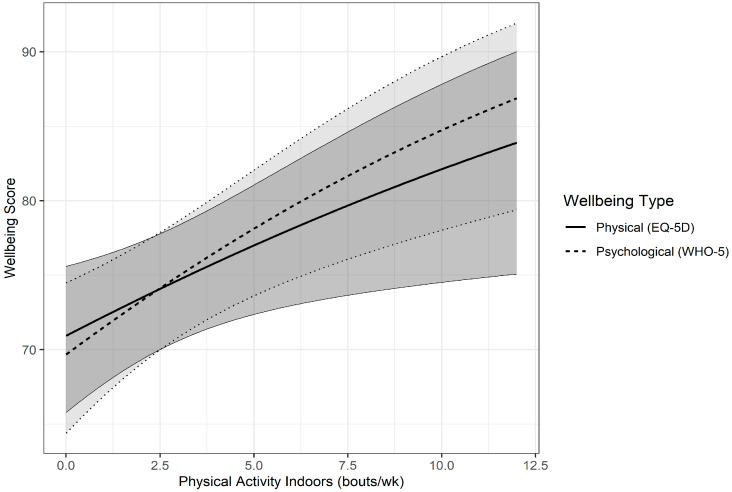
The association between the predictor variable, Physical Activity Indoors, and outcome variables, mean Physical (EQ-5D) and Psychological (WHO-5) Wellbeing scores, estimated from the GLMEM model. The grey areas designate the 95% confidence bands.

Physical activity indoors was also positively associated with physical wellbeing (z = 2.342, *p* = 0.0192) and psychological wellbeing (z = 3.322, *p* = 0.0009), with every one bout of PA indoors associated with a 1.22 unit increase in physical wellbeing score (η_p_^2^ of 0.018 (small effect size)) and a 2.11 unit increase in psychological wellbeing score (η_p_^2^ of 0.03 (small effect size) (Figure 2) (see Supplementary Materials Table S2 for complete GLMEM model and results)).

**Figure 3:**
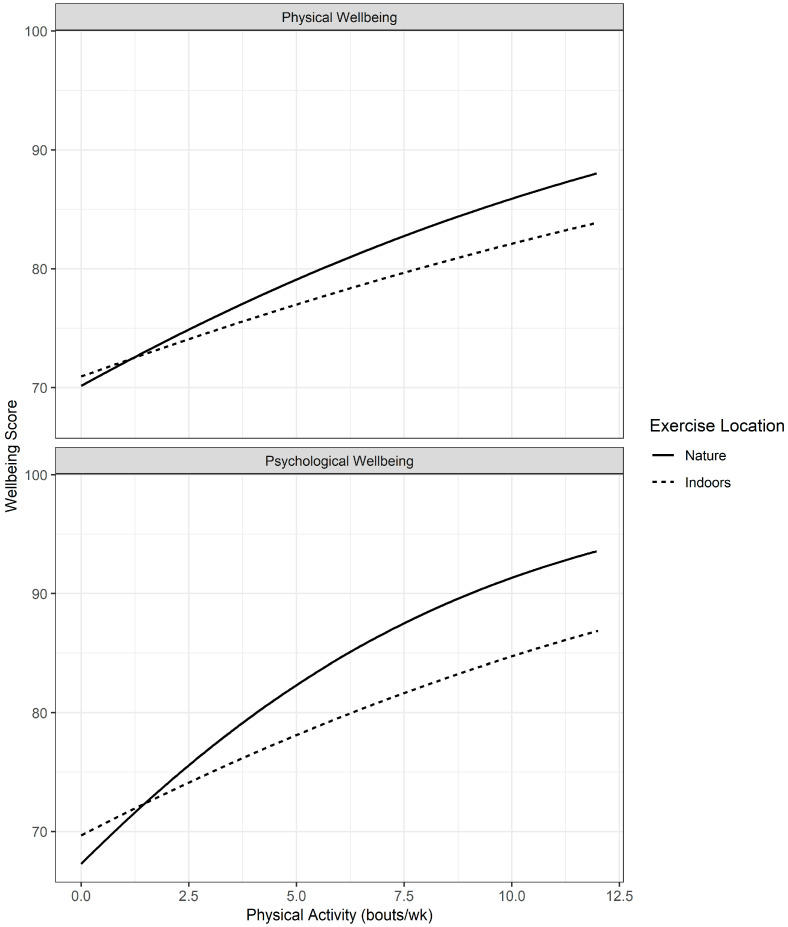
A comparison of the association between Physical Activity on Physical (EQ-5D) and Psychological (WHO-5) Wellbeing for different exercise locations.

Figure 3 shows a comparison of trend effects on physical and psychological wellbeing from PA in nature and indoors. For physical wellbeing, there was a significant difference, with PA in nature having a greater effect (Nature minus Indoors, z = 2.65, *p* = 0.008). For psychological wellbeing, there was also a significantly greater effect of PA in nature than indoors (Nature minus Indoors, z = 3.282, *p* = 0.001).

Physical activity outdoors in a built environment was not correlated with wellbeing (ChiSq(1) = 2.85, *p* = 0.09).

### 3.5. Nature-Relatedness

A majority of the respondents agreed with all questions regarding nature-relatedness, a construct to assess the level of connectedness an individual has with the natural world (Table 4). The average NR-6 score (on a scale from 1 to 5, where the higher the score the greater the nature-relatedness) from the six questions was 3.9 ± 0.7.

### 3.6. Nature-Relatedness and Wellbeing

In the GLMEM model, a significant interaction of nature-relatedness and PA in nature on wellbeing was identified (ChiSq(1) = 14.164, *p* = 0.0002), which shows there is a statistically significant difference in the association between nature-relatedness and both physical and psychological wellbeing depending on the degree of nature-relatedness, such that there was a greater effect of PA nature on wellbeing in those with greater nature-relatedness.

### 3.7. Nature-Relatedness and PA in Nature

The quantile regression model showed that there is a statistically significant association between nature-relatedness and physical activity in nature (t = 5.21, *p* ≤ 0.0001). Every one unit increase in nature-relatedness is associated with an estimated 0.98 unit increase in median physical activity in nature (see Supplementary Materials Table S3 for complete quantile regression model and results).

### 3.8. Connection to Place

Most respondents (64%) had a “deep sense of connection and identified with a place” that was special to them, although only 38% had a familial/ancestral connection to that place. Just over half (52%) “knew history and/or ancestral stories” of their special place. Amongst those who knew history or stories of their place, 74% declared an increased sense of guardianship for this place, 52% declared that knowing this made them feel safer, and 78% declared that knowing this altered their behaviour with respect to the environment.

### 3.9. Connection to Place and Frequency of PA in Nature

The quantile regression model showed that there was no statistically significant association between connection to place and PA in nature (t = 0.26, *p* = 0.79) (see Supplementary Materials Table S3 for complete quantile regression model and results).

### 3.10. Connection to Place and Wellbeing

In the GLMEM model, there was a significant interaction of connection to place and PA in nature on wellbeing (ChiSq(1) = 17.7702, *p* < 0.0001) with an inverse correlation, indicating that, compared to those without a connection to place, there was a lesser effect of PA in nature on wellbeing in those with a connection to place.

## 4. Discussion

Monitoring measures of perceived wellbeing and the environment in which individuals are physically active provides insight into the possible synergistic effects of physical activity and exposure to nature. The uniqueness of our study lies in the combination of measures relating not only to exposure to but also relatedness to the natural environment and wellbeing.

Our findings support those of Pasanen et al. [26], who found, in particular, enhanced emotional wellbeing in those exercising in natural environments. The present study expanded on this earlier work by also examining an individual’s affinity for nature and importance of place.

### 4.1. Physical Activity, Nature and Wellbeing

Early work with older people in Japan brought to light the impact one’s neighbourhood environment can have on longevity, in particular walkable green spaces being associated with longevity [18]. In another study, mortality was lower for a given level of income deprivation when more green space was present [50]. Maas et al. [51] found a number of specific physical and psychological disease states were reduced in those living in a greener local environment. Since these earlier studies, an association between (urban) green spaces and various aspects of wellbeing has been demonstrated in a number of (but not all) more recent studies [9]. In a large study of monozygotic twin adults, access to green space was inversely correlated with depression and stress [52]. In New Zealand, greater green space accessibility was also found not only to be related to reduced risk of mental health disorders but also to a reduced risk of cardiovascular disease [12].

In the present study, a stronger positive association between frequency of PA in nature and reported psychological wellbeing than with physical wellbeing was observed. Support for this finding can be found in a systematic review of 25 studies of health effects of activities in natural versus “synthetic” man-made environments [53]. In their systematic review, there was stronger evidence for improved emotional wellbeing with natural environment exposure than for physiological wellbeing as in our findings. It should be noted that the studies reviewed varied widely, and activities ranged from light gardening to strenuous exercise. In the present study, there was a measure of frequency (of bouts >20 min) but not duration nor intensity, and thus, load was not quantified. Since load (volume x intensity) is the primary determinant of the physiological response and adaptation, it may be that the reduced strength of association between physical wellbeing and PA in nature may be due to this source of variability.

Bowler et al. [46] posed that the health-promoting effect of nature may be more an effect of the environment to encourage people being there and doing particular activities. Hartig [54] suggested that there is an intertwining of environment and PA, such that green spaces are attractive for PA because of an association with restoration, and it is this that makes them attractive places for walking [55]. Even the effects of green space on psychological wellbeing may be indirect. Annerstedt et al. [56] found that having access to green space was not directly associated with mental health status, but they did find a synergistic effect of some qualities of green space in interaction with PA to reduce poor mental health. In a large survey in the U.K., a positive association between self-reported health and wellbeing and time spent in nature was observed, with those spending > 120 min in the last week in nature rating their health and wellbeing greater than those having had no nature contact [57]. Although PA was included in their model, the authors stated that the effects of PA were unable to be fully untangled and that nature contact was a “proxy” for PA. Some authors find that, although green space increases the likelihood of meeting PA recommendations, associations with health outcomes are not completely mediated by PA [12]. Thus, other benefits of just being in or near a natural environment are evidenced. These findings are supported by a study on forest bathing (Shinrin-yoku) in Japan in which one-half hour, half sitting, half walking, in a cityscape was compared with that in a forest. A number of physiological measures of stress, including cortisol and sympathetic drive, were measured and found to be lower with forest bathing [58]. In a lunchtime workplace intervention [59], park walks (versus in-office relaxation exercises or control) gave the greatest positive response in psychological outcomes, supporting the idea of a synergistic effect of PA and nature.

In a survey of forest and park users [60], reduced stress and headaches were reported after compared to before their park/forest visit. They found no difference between the two settings but did find greater improvement in those who performed higher intensity sports there (e.g., jogging, biking, playing ball) than in those engaged in less strenuous activities (e.g., taking a walk or relaxing) and that more people undertook more sports in and at the edge of forests. In another study, walking at a self-selected pace outdoors was faster than when conducted indoors, yet the perceived exertion was lower outdoors [61]. Synthesising information from these two studies and those of Hartig [54,62] and Annerstedt [56] provides insight into the present findings of enhanced psychological and physical wellbeing when physically active in nature.

The type or quality of green space may influence the response. An enhanced positive effect on psychological wellbeing has been observed in environments with greater biodiversity [63]. In the present study, however, we did not observe effects of PA in the various subcategories of natural environments, Nature-Constructed, Nature-Wilderness/Native Bush or Nature-Water, on wellbeing. It is probable that we were underpowered to observe any possible (differential) effects. For a broad overview of literature on “green exercise”, i.e., exercise in natural environments, the reader is directed to Gladwell et al. [64].

### 4.2. Physical Activity Indoors Versus in Nature

In the current study, PA indoors was the most frequently conducted. Unexpectedly, psychological wellbeing was also correlated with frequency of indoor PA, more so than physical wellbeing (health). This is in contrast to Pasanen et al. [26], who found that indoor PA was correlated with “general health” more so than “emotional wellbeing”, with PA in nature the inverse. This might also be due to the inability to accurately measure exercise load and the much greater power in their study (*n* = 2070). Our finding of a stronger correlation of PA in nature with psychological than physical wellbeing, however, agrees with that of Pasanen et al. [26]. Our results uphold our hypothesis that PA in nature is correlated with both physical and psychological wellbeing to a greater extent than in other environments.

### 4.3. Connections to Nature and Place

The concept of biophilia, our affinity for nature, is considered to be innate [19,20]; however, it may be that this construct relies on experiences in nature to nurture it [65,66]. Despite the fact that there is growing evidence that being in or close to nature facilitates PA, wellbeing and longevity, there is evidence of increased alienation from nature, termed the “extinction of experience” [67] or “nature deficit disorder” [68], especially amongst young people. It is posed that a reduction in nature experiences is responsible for a reduction in nature-relatedness/connectedness [64] and may impact PA.

In the present study, we found nature-relatedness to positively influence the associated between PA in nature and both physical and psychological wellbeing. This supports Mayer et al. [69], who found, in particular, better emotional wellbeing when individuals walked in nature versus in an urban environment or a virtual nature environment. They also measured nature-connectedness and, from their analyses, concluded that nature-connectedness mediates the effects of nature on wellbeing.

Connection to a particular place (topohilia) is also considered by some to be a fundamental human need [29] and is supported by the fact that over 60% of the respondents in the present study expressed a deep connection with a particular place, despite the fact that only just over one-third stated that they had a familial or ancestral connection to that place. Surprisingly, amongst those without familial ties, many knew traditional stories of the place, despite the lack of an ancestral link. The importance of a sense of place and belonging to environmentalism is exemplified by the large proportion of those who had this connection to place, stating that it enhanced their sense of guardianship and altered their behaviour. Nevertheless, in the present study, connection to place was not associated with wellbeing and was inversely correlated with physical activity in nature. It may be that place connectedness, in the New Zealand context, includes locations that are not necessarily conducive to physical activity.

That nature-relatedness was correlated with PA in nature and enhanced psychological and physical wellbeing also upholds our second hypothesis; however, that connection to place was not positively associated with PA in nature or wellbeing. Further research is warranted to confirm this finding and explore the divergent effects of nature-relatedness (biophilia) and connection to place (topophilia).

## 5. Conclusions and Implications

Physical activity in any environment is beneficial to wellbeing, both physical and psychological. However, physical activity in natural environments was associated with wellbeing, both physical and psychological, and although PA indoors was also associated with wellbeing, the correlation was not as strong as that conducted in nature. Having affinity for nature (nature-relatedness) also enhanced the association between PA in nature and wellbeing. Our results support the theory of synergistic benefits of PA in a natural environment. Having access to natural environments may improve health by stimulating one to be physically active and likely less sedentary. This has important implications for the design of new housing, especially as changes to the environment can significantly impact PA.

## Figures and Tables

**Table 1:** Sociodemographic characteristics of respondents (*n* = 179).

Age (years)	
mean ± SD	35 ± 17
range	18–83
Gender (*n* (%))	
Male	84 (47%)
Female	94 (52%)
Prefer not to say	1 (<1%)
Ethnicity (*n* (%))	
NZ European/European	143 (80%)
Māori	10 (6%)
Samoan	3 (2%)
Cook Islands Māori	1 (<1%)
Tongan	2 (1%)
Chinese	3 (2%)
Indian	3 (2%)
Other	14 (8%)
Marital status	
Married/living with partner	65 (37%)
Single/living alone	114 (64%)
Highest educational level obtained	
Secondary	57 (32%)
Tertiary (undergraduate qualification)	76 (42%)
Postgraduate qualification	46 (26%)
Employment status ^a^	
Unemployed	13 (6%)
Part-time employment	57 (27%)
Full-time employment	72 (34%)
Part-time unpaid employment	6 (3%)
Full-time unpaid employment	0 (0%)
Part-time studying	7 (3%)
Full-time studying	51 (24%)
Retired	7 (3%)
Physical disabilities that limit physical activity	
Yes	15 (8%)
No	164 (92%)

^a^ Respondents could choose multiple answers.

**Table 2:** Physical activity status from IPAQ short form (*n* = 179).

Physical Activity Level	*n* (%)	MET-Minutes Per Week (Mean ± SD)
Low	18 (10%)	194 ± 320
Moderate	66 (37%)	1834 ± 732
High	95 (53%)	7256 ± 5512

**Table 3:** Average weekly physical activity bouts * per environment (*n* = 179).

Environment	Frequency (Mean ± SD)
Indoors	2.3 ± 2.3
Outdoors Built Setting	1.6 ± 2.0
Nature	1.9 ± 3.1
Nature-Constructed	0.9 ± 1.8
Nature-Wilderness/Native Bush	0.8 ± 1.8
Nature-Water	0.3 ± 0.6

* 20 min or more.

**Table 4:** Nature-relatedness (NR-6) responses (*n* = 179).

	Strongly Agree/Agree (5–4) *n* (%)	Neither Agree nor Disagree (3) *n* (%)	Disagree/Strongly Disagree (2–1) *n* (%)	Average Score * Mean (SD)
My ideal vacation spot would be remote, wilderness	118 (66%)	25 (14%)	36 (20%)	3.6 (1.1)
I think how my actions affect the environment	145 (81%)	26 (14%)	8 (4%)	3.9 (0.7)
My connection to nature/environment is part of my spirituality	105 (59%)	41 (23%)	33 (18%)	3.7 (1.2)
I take notice of wildlife wherever I am	155 (87%)	20 (11%)	4 (2%)	4.3 (0.8)
My relationship to nature is an important part of who I am	121 (68%)	48 (27%)	10 (6%)	4.0 (1.0)
I feel connected to all living things and the earth	110 (62%)	40 (22%)	29 (16%)	3.7 (1.1)

* On a scale from 5 (strongly agree) to 1 (strongly disagree).

## Data Availability

The raw data supporting the conclusions of this article will be made available by the authors on request.

## Supplementary Materials

The following supporting information can be downloaded at: https://www.mdpi.com/article/10.3390/ijerph22020299/s1, Supplementary File S1: Survey: Experiences in Nature and Hauora (Health); Supplementary Table S1: Survey Results: Perceived Psychological Wellbeing from WHO-5 (*n* = 179); Supplementary Table S2: Analysis of Deviance Table for the Health Model; Supplementary Table S3: Summary Table for the Physical Activity in Nature Model.
