# Supplementary material for: The Physical Activity Environment, Nature-Relatedness and Wellbeing
Source: Int J Environ Res Public Health. 2025 Feb 17;22(2):299. doi: 10.3390/ijerph22020299 (PMC11855637; doi:10.3390/ijerph22020299)
Supplement: Supplementary file 1 [file ijerph-22-00299-s001.zip › Supplementary Materials 1 Survey.pdf]

# Experiences in Nature and Hauora

---

**Start of Block: Default Question Block**

Q1.1 Experiences in Nature and Hauora (Health)
https://otago.au1.qualtrics.com/jfe/form/SV_aeYz89KIIhHkhMO

I have read and understood the information sheet (below) concerning this project. I understand that I am free to withdraw from the survey at any stage without consequence. By participating in this survey, I know that:- My participation in the project is entirely voluntary; I have to complete the survey and meet the participant criteria outlined in the information sheet to be eligible for the prize draw; I do not have to enter the prize draw, but if I do, I am to provide contact details; Personal identifying information (contact details) will be destroyed at the conclusion of the project but any raw data on which the results of the project depend will be retained in secure storage for at least five years; The results of the project may be published and will be available in the University of Otago Library (Dunedin, New Zealand) but every attempt will be made to preserve my anonymity. By clicking the next button, I agree to take part in this project.

**End of Block: Default Question Block**

---

**Start of Block: General Information**

Q56 Do you presently live in New Zealand?

- ○ Yes (1)
- ○ No (2)

*Skip To: End of Survey If Do you presently live in New Zealand? = No*

---

Q57 Are you 18 years of age or older?

- ○ Yes (1)
- ○ No (2)

*Skip To: End of Survey If Are you 18 years of age or older? = No*

---

Q2.1 How old are you?

| | 18 26 34 43 51 59 67 75 84 92 100 |
|---|---|
| Slide bar to your age () | |

Q2.2 Are you?

- ◯ Male (1)
- ◯ Female (2)
- ◯ Non-binary / third gender (3)
- ◯ Prefer not to say (4)

Q2.3 What ethnic group do you predominantly belong to?

- ◯ New Zealand/European (1)
- ◯ Māori (2)
- ◯ Samoan (3)
- ◯ Cook Islands Maori (4)
- ◯ Tongan (5)
- ◯ Niuean (6)
- ◯ Chinese (7)
- ◯ Indian (8)
- ◯ Other, e.g., Dutch, Japanese, Tokelauan (Please enter below) (9)

________________________________________________

Q2.4 Marital Status

- ○ Married/living with partner (1)
- ○ Single/living alone (2)

Q2.5 Highest Education Level Obtained

- ○ Secondary (1)
- ○ Tertiary (Undergraduate Qualification) (2)
- ○ Postgraduate Qualification (3)

Q2.6 Employment Status (may choose more than one)

- ☐ Unemployed (1)
- ☐ Part-Time Employment (2)
- ☐ Full-Time Employment (3)
- ☐ Part-Time Unpaid Employment (e.g., Volunterring) (4)
- ☐ Full-Time Unpaid Employment (e.g., Volunteering) (5)
- ☐ Part-Time Studying (6)
- ☐ Full-Time Studying (7)
- ☐ Retired (8)

Q2.7 Any Physical Disability that Limits Physical Activity?

○ Yes (1)

○ No (2)

---

Q2.8 What is your Postcode?

________________________________________________________________

**End of Block: General Information**

---

**Start of Block: Nature Relatedness**

Q3.1 **Nature Relatedness Scale (NR-6)** For each of the following, please rate the extent to which you agree with each statement, using the scale from strongly agree to strongly disagree

as shown below. Please respond as you really feel, rather than how you think “most people” feel.

| | Strongly Agree (1) | Somewhat agree (2) | Neither agree nor disagree (3) | Somewhat disagree (4) | Strongly disagree (5) |
|---|---|---|---|---|---|
| My ideal vacation spot would be a remote, wilderness area (1) | ○ | ○ | ○ | ○ | ○ |
| I always think about how my actions affect the environment (2) | ○ | ○ | ○ | ○ | ○ |
| My connection to nature and the environment is a part of my spirituality (3) | ○ | ○ | ○ | ○ | ○ |
| I take notice of wildlife wherever I am (4) | ○ | ○ | ○ | ○ | ○ |
| My relationship to nature is an important part of who I am (5) | ○ | ○ | ○ | ○ | ○ |
| I feel very connected to all living things and the Earth (6) | ○ | ○ | ○ | ○ | ○ |

**End of Block: Nature Relatedness**

---

**Start of Block: Perceived General Health**

Q4.2 Please indicate on the Visual Analogue Scale below how good or bad your own health state is today
The best health state you can imagine is marked 100 and the worst health state you can imagine is 0
**Please move the scale from 0 to 100 indicating how good or bad your health state is today**

| | Worst Imaginable health | Best imaginable health |
| --- | --- | --- |
| | 0 10 20 30 40 50 60 70 80 90 100 | |
| Health State Today () | | |

**End of Block: Perceived General Health**

**Start of Block: Emotional Wellbeing Scale**

Q5.1 **General Well-Being** Please select an answer for each of the five statements that is closest to how you have been feeling over the last two weeks.

| | Strongly agree (1) | Somewhat agree (2) | Neither agree nor disagree (3) | Somewhat disagree (4) | Strongly disagree (5) |
| --- | --- | --- | --- | --- | --- |
| I feel cheerful and in good spirits (1) | o | o | o | o | o |
| I feel calm and relaxed (2) | o | o | o | o | o |
| I feel active and vigorous (3) | o | o | o | o | o |
| I wake up feeling fresh and rested (4) | o | o | o | o | o |
| My daily life is filled with things that interest me (5) | o | o | o | o | o |

**End of Block: Emotional Wellbeing Scale**

---

**Start of Block: Typical Weekly Physical Activity**

Q6.1 **Estimate in a typical week how many times you are physically active for 20 minutes or longer?** (e.g. exercise that made you breath somewhat harder than normal)

________________________________________________________________

*Skip To: End of Block If Condition: Estimate in a Typical Week:... Is Equal to 0. Skip To: End of Block.*

---

Q6.2 **What percent of your weekly physically active time is in each type of location?** (Locations where PA can take place)
(**This should total up to 100%**)

○ **Indoors** (This includes indoor sports settings (e.g. sports hall, gym, swimming pool) and indoors at home or workplace (e.g. heavy lifting, other heavy house work, home cycle trainer, treadmill, gymnastics): (1)

________________________________________________

○ **Outdoors in a Built Setting** (This includes sport and non- sport specific locations such as streets, cycle lanes, sports fields, outdoor swimming pool, basketball court, sport fields (e.g. rugby, cricket grounds): (2)

________________________________________________

○ **Nature – Constructed** (This includes natural environments that are to a large extent constructed e.g. formal gardens, exotic pine forests, farmers paddocks, ski fields): (3)

________________________________________________

○ **Nature – Wilderness/Native Bush** (This includes most D.O.C. land, wilderness and other native bush areas): (4)

________________________________________________

○ **Nature – Water** (This includes in or on the ocean, lakes, rivers or streams): (6)

________________________________________________

---

Q6.3 **What % of your time spent being physically active is with one or more person(s)?** (e.g. all physical activity that is not by yourself)

0 10 20 30 40 50 60 70 80 90 100

| | |
|---|---|
| Estimate () | |

Q6.4 **What % of your time spent being physically active, is with an animal?** (e.g. dog, horse)

| | 0 | 10 | 20 | 30 | 40 | 50 | 60 | 70 | 80 | 90 | 100 |
|---|---|---|---|---|---|---|---|---|---|---|---|
| Estimate () | | | | | | | | | | | |

**End of Block: Typical Weekly Physical Activity**

**Start of Block: Time in Nature Not Physically Active**

Q7.1 **Estimate in a Typical Week:**

How often do you spend time in nature or looking at nature, not engaged in strenuous PA? (Examples: sitting on a bench at the park, chilling on the beach, driving through woods, sitting in car at the beach)

- ○ How many times per week? (1) ________________________________________________

- ○ Average duration? (Minutes) (2) ________________________________________________

*Skip To: End of Block If Condition: How many times per week? Is Equal to 0. Skip To: End of Block.*

Q51 What % of your time spent inactive, in nature, is with one or more person?

| | 0 | 10 | 20 | 30 | 40 | 50 | 60 | 70 | 80 | 90 | 100 |
|---|---|---|---|---|---|---|---|---|---|---|---|
| Estimate () | | | | | | | | | | | |

Q52 What % of your time spent inactive, in nature, is with an animal (e.g. dog, horse)?

| | 0 10 20 30 40 50 60 70 80 90 100 |
| --- | --- |
| Estimate () | |

**End of Block: Time in Nature Not Physically Active**

**Start of Block: IPAQ**

Q8.1 We are interested in finding out about the kinds of physical activities that people do as part of their everyday lives. The questions will ask you about the time you spent being physically active in the **last 7 days**. Please answer each question even if you do not consider yourself to be an active person. Please think about the activities you do at work, as part of your house and yard work, to get from place to place, and in your spare time for recreation, exercise or sport.

---

Q8.2 Think about all the **vigorous activities** that you did in the **last 7 days**. Vigorous physical activities refer to activities that take hard physical effort and make you breathe much harder than normal. Think only about those physical activities that you did for **at least 10 minutes at a time**. **(Answer 0 if you have not done any vigorous physical activity)**

○ Days Per Week (1) ________________________________________________

*Skip To: Q8.4 If Condition: Days Per Week Is Equal to 0. Skip To: Think about all the moderate activiti....*

---

Q8.3 How much time did you usually spend doing **vigorous** physical activities on one of those days?

○ Hours Per Day (1) ________________________________________________

○ Minutes Per Day (2) ________________________________________________

---

Q8.4 Think about all the **moderate activities** that you did in the **last 7 days**. Moderate activities refer to activities that take moderate physical effort and make you breathe somewhat harder than normal. Think only about those physical activities that you did for **at least 10 minutes at a time**. During the **last 7 days**, on how many days did you

do **moderate** physical activities like carrying light loads, bicycling at a regular pace, or doubles tennis?  Do not include walking.   **(Answer 0 if you have not done any moderate physical activity)**

○ Days per week  (1) ________________________________________________

*Skip To: Q8.6 If Condition: Days per week Is Equal to 0. Skip To: Think about the time you spent walkin....*

Q8.5 How much time did you usually spend doing **moderate** physical activities on one of those days?

○ Hours Per Day  (1) ________________________________________________

○ Minutes Per Day  (2) ________________________________________________

Q8.6 Think about the time you spent **walking** in the **last 7 days**.  This includes at work and at home, walking to travel from place to place, and any other walking that you have done solely for recreation, sport, exercise, or leisure.     During the **last 7 days**, on how many days did you **walk** for at least 10 minutes at a time?
**(Answer 0 if you have done no walking)**

○ Days Per Week  (1) ________________________________________________

*Skip To: Q8.8 If Condition: Days Per Week Is Equal to 0. Skip To: The last question is about the time y....*

Q8.7 How much time did you usually spend **walking** on one of those days?

○ Hours Per Day  (1) ________________________________________________

○ Minutes Per Day  (2) ________________________________________________

Q8.8 The last question is about the time you spent **sitting** on weekdays during the **last 7 days**.  Include time spent at work, at home, while doing course work and during leisure time.  This may include time spent sitting at a desk, visiting friends, reading, or sitting or lying

down to watch television. During the **last 7 days**, how much time did you spend **sitting** on a **week day**?

○ Hours Per day (1) ________________________________________________

○ Minutes Per Day (2) ________________________________________________

**End of Block: IPAQ**

---

**Start of Block: Connection to Place (Matauranga Maori)**

Q9.1 Tūrangawaewae means a place to stand, are places where we feel especially connected to the taiao (nature). They are our foundation, our place in the world, where a person feels strong, safe and at home. **Do you have a deep sense of connection and identify with, a place that is special to you (tūrangawaewae)?**

○ Yes (1)

○ No (2)

*Skip To: End of Block If Tūrangawaewae means a place to stand, are places where we feel especially connected to the taiao... = No*

Q9.2 **Do you have a familial/ancestral connection (whakapapa) to this place?**

○ Yes (1)

○ No (2)

Q9.3 **Do you know any history and/or ancient stories (pūrākau) from this place?**

○ Yes (1)

○ No (2)

*Skip To: End of Block If Do you know any history and/or ancient stories (pūrākau) from this place? = No*

Q9.4 **Does knowing the history/ancient stories (pūrākau) from this place give you a sense of greater sense of guardianship (kaitiakitanga) for this place?**

- ○ Yes (1)
- ○ No (2)

---

Q53 **Does knowing the history/ancient stories (pūrākau) or ancestors/environmental signs (atua) associated with this place make you feel safer?**

- ○ Yes (1)
- ○ No (2)

---

Q9.5 **Does knowing the history/ancient stories (pūrākau) from this place alter your behaviour with respect to the environment (taiao)?**

- ○ Yes (1)
- ○ No (2)

**End of Block: Connection to Place (Matauranga Maori)**

---

**Start of Block: Sensing Nature**

Q10.1 When in nature, either active or inactive, which, if any, of the following evoke strong feelings and/or appreciation for this place/the environment? (This could be physical, emotional or spiritual i.e. Tinana, Hinengaro or Wairua)

- ▢ Sight (1)
- ▢ Smell (2)
- ▢ Sounds (3)
- ▢ Touch (4)

Q10.2 Rate these senses from very important (20) to not at all important (0) in your opinion (You may give the same rating for all if you feel they are of equal importance)

| | 0 2 4 6 8 10 12 14 16 18 20 |
|---|---|
| Sight () | |
| Smell () | |
| Sounds () | |
| Touch () | |

Q10.3 Senses can bring up memories of a particular place. Which, if any, of these bring up memories of a particular place, when somewhere else?

- ☐ Sight (1)
- ☐ Smell (2)
- ☐ Sounds (3)
- ☐ Touch (4)
- ☐ None (5)

*Skip To: End of Block If Senses can bring up memories of a particular place. Which, if any, of these bring up memories of... = None*

Carry Forward Selected Choices from "Senses can bring up memories of a particular place. Which, if any, of these bring up memories of a particular place, when somewhere else?"

Q10.4 Of those senses that bring up memories of a particular place, rate them from very important (20) to not at all important (0) in your opinion

0 2 4 6 8 10 12 14 16 18 20

| | |
|---|---|
| Sight () | |
| Smell () | |
| Sounds () | |
| Touch () | |
| None () | |

Q45 If you'd like to learn more about a Maori world view of environmental science and health please visit https://toitangata.co.nz/our-mahi/atua-matua/

**End of Block: Sensing Nature**

**Start of Block: Block 10**

Q50 Would you like to be entered into the prize draw?

- Yes (1)
- No (2)

*Skip To: End of Survey If Would you like to be entered into the prize draw? = No*

Q49 This is a link to the prize draw, please click next on this page once you have copied/opened the link so your response can be recorded!

https://otago.au1.qualtrics.com/jfe/form/SV_2lDTRBfKJTtadsG

**End of Block: Block 10**
