# Supplementary material for: The Physical Activity Environment, Nature-Relatedness and Wellbeing
Source: Int J Environ Res Public Health. 2025 Feb 17;22(2):299. doi: 10.3390/ijerph22020299 (PMC11855637; doi:10.3390/ijerph22020299)
Supplement: Supplementary file 1 [file ijerph-22-00299-s001.zip › Supplementary Materials Table S1.pdf]

Table S1. Perceived psychological wellbeing from WHO-5 (n=179)

	**Study Sample** (n = 179)			
	Strongly agree / Agree n (%)	Neither agree nor disagree n (%)	Strongly disagree / Disagree n (%)	Average (Mean ± SD)
**Psychological Wellbeing**				
I feel cheerful and in good spirits	139 (77.7%)	23 (12.8%)	17 (9.5%)	3.9 ± 0.9
I feel calm and relaxed	128 (71.5%)	31 (17.3%)	20 (11.2%)	3.8 ± 0.9
I feel active and vigorous	106 (59.2%)	38 (21.2%)	35 (19.6%)	3.5 ± 1.1
I wake up feeling fresh and rested	92 (51.4%)	37 (20.7%)	50 (28%)	3.3 ± 1.1
My daily life is filled with things that interest me	125 (70%)	38 (21.2%)	16 (9%)	3.9 ± 0.9
