# Supplementary material for: The Physical Activity Environment, Nature-Relatedness and Wellbeing
Source: Int J Environ Res Public Health. 2025 Feb 17;22(2):299. doi: 10.3390/ijerph22020299 (PMC11855637; doi:10.3390/ijerph22020299)
Supplement: Supplementary file 1 [file ijerph-22-00299-s001.zip › Supplementary Materials Table S2.pdf]

```
> Anova(Mod_3PA_2way_2.1, type="II")
Analysis of Deviance Table (Type II Wald chisquare tests)

Response: Health_Score
                              Chisq Df Pr(>Chisq)
GENDER                       0.0432  1  0.8353762
AGE2                         5.8389  1  0.0156753 *
Disability                   8.1610  1  0.0042800 **
NR_Ave_invert2               2.2317  1  0.1352008
Health_Variable              4.7221  1  0.0297777 *
CONNECT2                     0.0421  1  0.8374917
TotalPA_pWk_MET2             0.9266  1  0.3357443
PA_Nat2                      3.3541  1  0.0670383 .
PA_IN2                      11.9245  1  0.0005540 ***
PA_Built2                    2.8583  1  0.0909030 .
AGE2:CONNECT2                8.2331  1  0.0041134 **
Disability:NR_Ave_invert2    4.2987  1  0.0381418 *
Disability:Health_Variable   1.7370  1  0.1875231
NR_Ave_invert2:PA_Nat2      14.1640  1  0.0001675 ***
Health_Variable:PA_Nat2      7.8339  1  0.0051277 **
CONNECT2:PA_Nat2            17.7702  1  2.493e-05 ***
TotalPA_pWk_MET2:PA_Built2   4.1810  1  0.0408786 *
Health_Variable:PA_IN2       0.6742  1  0.4115895
---
Signif. codes:  0 '***' 0.001 '**' 0.01 '*' 0.05 '.' 0.1 ' ' 1
```
