# Supplementary material for: The Physical Activity Environment, Nature-Relatedness and Wellbeing
Source: Int J Environ Res Public Health. 2025 Feb 17;22(2):299. doi: 10.3390/ijerph22020299 (PMC11855637; doi:10.3390/ijerph22020299)
Supplement: Supplementary file 1 [file ijerph-22-00299-s001.zip › Supplementary Materials Table S3.pdf]

```
> mod_nr2 <- rq(PA_Nat2 ~ NR_Ave_invert + CONNECT2 + AGE2 + GENDER + Disability, tau=0.5, method="fn", data=data_m1)
> summary(mod_nr2, se="boot", R=1000)

Call: rq(formula = PA_Nat2 ~ NR_Ave_invert + CONNECT2 + AGE2 + GENDER +
    Disability, tau = 0.5, data = data_m1, method = "fn")

tau: [1] 0.5

Coefficients:
              Value    Std. Error t value  Pr(>|t|)
(Intercept)   -4.60576  0.69000   -6.67497  0.00000
NR_Ave_invert  0.98141  0.18838    5.20971  0.00000
CONNECT2Yes    0.06523  0.24703    0.26405  0.79206
AGE2           0.36538  0.20907    1.74763  0.08231
GENDER2       -0.33884  0.25212   -1.34398  0.18073
Disability2    0.25618  0.51118    0.50116  0.61690
```
